# Conviction in the absence of proof: Conspiracy mentality mediates religiosity’s relationship with support for COVID-19 conspiracy narratives

**DOI:** 10.3389/fpsyg.2023.1026144

**Published:** 2023-02-16

**Authors:** Hilmar Grabow, Anne Rock

**Affiliations:** Social and Political Psychology, Kiel University, Kiel, Germany

**Keywords:** religiosity, conspiracy mentality, conspiracy narratives, COVID-19, critical thinking

## Abstract

The belief in conspiracy narratives and the concept of conspiracy mentality have gained increasing attention in psychological science over the last years. A cornerstone is the assumption of secretly acting groups pulling the strings in world affairs. Based on the reasoning that religiosity and conspiracy mentality share a common core – both can be understood as strong convictions without final proof or even in the face of contradictory evidence – we hypothesised that the support of COVID-19 conspiracy narratives would be related to religiosity as well as conspiracy mentality. Given that religious socialisation usually starts very early in life, we furthermore assumed that religiosity could be an antecedent of conspiracy mentality. Therefore, we tested a mediation model comprising religiosity (predictor), support of conspiracy narratives (criterion), and conspiracy mentality (mediator) among *N* = 616 participants of an online survey. Analyses revealed significant total and indirect effects, supporting our hypothesis.

## 1. Introduction

The enduring COVID-19 pandemic has reminded us once again of humans’ extraordinary capability to develop and maintain strong convictions based on rumours, hearsay, or unfunded arguments – even in the face of stringent, compelling counterevidence. More often than never such “arguments” are embedded in conspiracy narratives, i.e., assumptions about incidents happening in the world being steered by malevolent groups trying to reap benefits (Goertzel, 1994). According to Barkun (2013), such narratives typically feature three elements: nothing happens by chance, nothing is as it seems, and everything is connected. Conspiracy narratives are self-contained, believers appear to be immune to any kind of criticism or doubt and reject more plausible explanations or contradicting arguments; chance or chaos has no place (Wood et al., 2012).

Interestingly, there appears to be a tendency to generally support conspiracy narratives insofar as people who agree with one narrative are more likely to agree with others, too – regardless of inconsistencies or even obvious contradictions between them (Swami et al., 2011; Wood et al., 2012; Bartoschek, 2015). Bruder et al. (2013) introduce the concept of conspiracy mentality, an intrinsic tendency to believe in conspiracy narratives which is characterized by a general distrust in elites or supposedly powerful groups and the conviction that events do not unfold by chance but are controlled by these groups. Conspiracy mentality and conspiracy narratives, insofar, are closely related but not the same. We theorise that conspiracy mentality predicts the approval of conspiracy narratives. In a further step, we extend our model by including religiosity.

The study was conducted in Germany where Christianity has long been (Kinzig, 2003) and still is (e.g., Frerk, 2015) extremely influential on societal level and political level. Our concept of religiosity therefore heavily relies on the Christian variety. Given their similarities, e.g., featuring one almighty god, it may however well generalise to other Abrahamic religions. Religion can be understood as a multifaceted construct – is not limited to a more or less pronounced conviction of a god’s existence but also entails actions like attending services or praying. Hill and Hood (1999) offer an overview over numerous scales for the measurement of (different aspects of) religiosity. Indeed, there is a plethora of conceptualisations and dimensions of religiosity (e.g., Wulff, 1997). Vaas and Blume (2011) describe religion by seven features including the belief in supernatural power(s). This is the feature we focus on in this study because a conviction of god’s existence seems to be a (if not the) cornerstone of monotheistic religions and hence indispensable for our model. Religiosity, thus, entails the belief in the existence of an almighty god, in divine interventions and miracles, or in the absence of chance. God is believed in even though it is impossible for humans to prove his existence (Ratzinger, 2011). Parallels to conspiracy mentality are obvious as in both cases events are perceived as being controlled by a powerful entity – a group or a god – without (conclusive) evidence of their existence. Chance as a cause is ruled out.

Conspiracy mentality and religiosity have much in common but there are also differences. Despite ample evidence of the opposite (e.g., Hitchens, 2007), Christians usually believe in a benevolent god (“A theist normally holds that God is by nature morally perfectly good …” – as Swinburne, 1977, p. 184, puts it), while conspiracy mentality implies the conviction that a malevolent entity guides world affairs. While god is not only feared but also praised by followers and appealed to in the hope of help or guidance, malevolent groups would rather be dreaded for their (potential) negative impact on people. Thus, attitudes towards these different entities as well as related actions differ. This is why we consider conspiracy mentality and religiosity as two separate constructs despite their striking similarities.

The German verb *glauben* (to believe) is ambiguous: on the one hand, it qualifies statements as uncertain. “*Ich glaube, morgen wird es regnen*” (I think it will rain tomorrow) implies uncertainty – the speaker does not know for sure tomorrow’s weather conditions. The less sure a person is, the more emphasis would lie on the *glauben* component. Here, *glauben* is opposed to *wissen* (to know). On the other hand, *glauben* can emphasise certainty. “*Ich glaube an Gott*” (I believe in god) implies that the speaker is sure god exists – the more so the stronger they *glauben*. Here, *glauben* is very similar to *wissen*. Per definition by Ratzinger (2011), however, this knowledge is not scientifically grounded but implies the feature of exclusivity: only those who have turned to god can truly believe, others have no access to any proof for the belief’s plausibility, rendering it unfalsifiable.

It is this latter sense of the word *glauben* we tap into with our concept of religiosity: the stronger a person believes, the smaller their doubt, the larger their certainty in, e.g., the existence of god [this conception is very close to Dawkins’ (2006, p. 198) definition of faith: “It means blind trust, in the absence of evidence, even in the teeth of evidence”]. In this respect, religiosity entails convictions despite absence of proof or as in the case of believing in an omniscient, omnipotent, benevolent god even despite overwhelming evidence of the contrary (Law, 2010). Law (2011) also points out similarities between conspiracy narratives and religious statements. Thus, the conceptual overlap of religiosity and conspiracy mentality becomes obvious.

Summing up, we deal with three concepts: religiosity, conspiracy mentality, and conspiracy narratives. Given that religion usually is passed on from generation to generation (e.g., Myers, 1996; Dawkins, 2007) we hypothesise that religiosity is encouraged very early in life and precedes conspiracy mentality. That is why we suggest that religiosity predicts conspiracy mentality which, in turn, predicts (support of) conspiracy narratives. Moreover, we expect a direct relation of religiosity and conspiracy narratives. We tested this mediation model in the context of COVID-19 narratives.

## 2. Methods

### 2.1. Procedure

We conducted an online survey with a German-speaking convenience sample. Participants were recruited online *via* social media as well as offline *via* placards.

### 2.2. Sample

The sample (*N* = 616) comprises 295 female and 308 male participants, while 13 consider themselves diverse or indicated no gender; their age (*M* = 43.26, *SD* = 12.84) ranged from 19 to 81. Almost half of the participants (*N* = 286) possess an academic degree, 327 do not. Most participants (*N* = 396) did not affiliate themselves with any religious group, 189 with Christianity; 31 indicated other religious affiliations or did not answer.

### 2.3. Measures

The survey was conducted as part of a larger research project directed by the corresponding author. Therefore, the questionnaire contained additional measures relevant to other research questions of that project. However, all data analyses reported in the article are novel, and the findings have not been published elsewhere. Participants indicated their answers regarding the three constructs described below on a 7-point scale ranging from “I do not agree at all – 0” *via* “1,” “2,” “3,” “4,” “5” to “I completely agree – 6.”

The predictor, **religiosity,** was initially measured by 10 items mainly tapping into the belief in an intervening, almighty, omniscient god (e.g., “God exists,” “I have confidence in god‘s decisions,” “There is no such thing as chance – god directs,” or “My belief in god is enough for me as proof of his existence”). The only item not explicitly referring to god (“There is a life after death”) was dropped from the final scale for conceptual reasons. Moreover, this led to a slight increase in reliability (Cronbach’s α = 0.967).

To measure the mediator, **conspiracy mentality,** we used the conspiracy mentality scale by Imhoff and Bruder (2014), comprising items like “There are very many important things happening in the world that the public is never informed about,” “A few powerful groups of people determine the fate of millions of people,” or “There are secret organisations that have great influence on political decisions.” Given their comparably low fit two reverse-coded items (“I consider the various conspiracy theories circulating in the media to be utter nonsense” and “There is no reasonable reason to distrust governments, intelligence agencies, or the media”) were dropped, resulting in a 10-item scale (Cronbach’s α = 0.913).

The criterion, **conspiracy narratives,** was tailored to the current COVID-19 pandemic. On the one hand, we adopted four items previously used by Imhoff and Lamberty (2020), e.g., “Corona was deliberately brought into the world to reduce the population” and “Dark forces want to use the virus to dominate the world.” On the other hand, we developed five items inspired by social media or news reports, e.g., “The coronavirus is a bioweapon of the Asians” and “The coronavirus does not exist, it is a government invention to restrict our fundamental rights.” Overall, the 9-item scale appeared reliable: Cronbach’s α = 0.899. Please refer to the Supplementary material for a complete listing of all items in German (as employed in the study) and English.

## 3. Results

Table 1 contains the means and standard deviations of religiosity, conspiracy mentality, conspiracy narratives, and socio-demographic variables as well as their correlations. Unsurprisingly, religiosity is significantly stronger among the Christians in the sample (*M* = 2.10, *SD* = 1.84) than the participants not religiously affiliated (*M* = 0.25, *SD* = 0.66); *t*(583) = 17.89, *p* < 0.001. For conspiracy mentality, the difference is smaller (Christians: *M* = 1.71, *SD* = 1.26; non-affiliated: *M* = 1.38, *SD* = 1.04) yet significant: *t*(583) = 3.32, *p* = 0.001. Agreement with conspiracy narratives is generally low; the Christians’ average (*M* = 0.22, *SD* = 0.55) is not significantly higher than the non-affiliated participants’ (*M* = 0.16, *SD* = 0.52): *t*(583) = 1.34, *p* = 0.182).

**Table 1:** Means of and correlations between constructs and socio-demographic variables.

			Correlations				
	*N*	M (SD)	1	2	3	4	5	6	7
1 Religiosity	616	0.89 (1.52)							
2 Conspiracy mentality	616	1.53 (1.17)	0.264^***^						
3 Conspiracy narratives (CN)	616	0.19 (0.56)	0.275^***^	0.527^***^					
4 CN (power)	616	0.13 (0.46)	0.299^***^	0.456^***^	0.916^***^				
5 CN (disinformation)	616	0.29 (0.92)	0.206^***^	0.511^***^	0.916^***^	0.680^***^			
6 Age	616	43.26 (12.84)	−0.204^***^	−0.128^**^	−0.135^***^	−0.111^**^	−0.136^***^		
7 Gender^a^	603	48.92% women	0.168^***^	0.035	0.031	0.044	0.012	−0.192^***^	
8 Education^b^	613	46.66% with academic degree	−0.079^*^	−0.173^***^	−0.105^**^	−0.122^**^	−0.070	0.105^**^	−0.100^*^

^a^Men = 0, Women = 1. ^b^Non-academic = 0, Academic = 1. ^*^p < 0.05, ^**^p < 0.01, ^***^p < 0.001.

All 28 items capturing religiosity, conspiracy mentality, and conspiracy narratives were included in a principal component analysis (oblimin rotation with Kaiser normalisation, three fixed factors). Sampling adequacy was marvellous (Kaiser and Rice, 1974) – Kaiser–Meyer–Olkin (*KMO*) measure = 0.935; Bartlett’s test of sphericity significant: χ^2^(378) = 14,625.21, *p* < 0.001. The three factors collectively explained 65.84% of variance. The solution neatly represents the three factors intended to be measured (refer to Table 2).

**Table 2:** Principal component analyses: factor loadings.

	Pattern matrix			Structure matrix		
	1	2	3	1	2	3
Items: religiosity, conspiracy mentality, conspiracy narratives						
r01	−0.059	**−0.933**	−0.006	0.162	**−0.917**	0.213
r02	−0.024	**−0.923**	−0.007	0.194	**−0.916**	0.225
r03	−0.076	**−0.910**	0.022	0.151	**−0.898**	0.228
r04	−0.032	**−0.956**	−0.007	0.195	**−0.947**	0.231
r05	0.010	**−0.830**	0.147	0.272	**−0.871**	0.369
r06	0.101	**−0.799**	0.012	0.298	**−0.826**	0.265
r07	0.013	**−0.871**	−0.014	0.216	**−0.871**	0.220
r08	0.121	**−0.839**	−0.042	0.304	**−0.857**	0.230
r10	−0.006	**−0.959**	−0.060	0.199	**−0.942**	0.189
m01	**0.773**	−0.059	−0.076	**0.754**	−0.225	0.271
m02	**0.683**	0.049	−0.043	**0.653**	−0.103	0.237
m03	**0.767**	−0.028	−0.114	**0.725**	−0.182	0.222
m04	**0.781**	−0.045	0.043	**0.810**	−0.244	0.389
m06	**0.723**	−0.022	0.110	**0.775**	−0.224	0.425
m07	**0.644**	−0.013	0.280	**0.767**	−0.241	0.560
m09	**0.727**	−0.003	−0.026	**0.717**	−0.171	0.287
m10	**0.632**	−0.046	0.164	**0.713**	−0.240	0.447
m11	**0.810**	0.034	−0.018	**0.794**	−0.156	0.320
m12	**0.767**	0.016	0.117	**0.813**	−0.199	0.442
n01	−0.106	0.027	**0.866**	0.259	−0.175	**0.814**
n02	0.080	−0.130	**0.531**	0.339	−0.288	**0.599**
n03	−0.101	−0.037	**0.751**	0.230	−0.209	**0.718**
n04	−0.116	−0.006	**0.780**	0.220	−0.183	**0.732**
n05	0.165	0.015	**0.747**	0.482	−0.221	**0.814**
n06	0.166	0.065	**0.759**	0.476	−0.174	**0.814**
n07	0.162	0.081	**0.738**	0.459	−0.152	**0.786**
n08	0.093	−0.102	**0.706**	0.420	−0.309	**0.773**
n09	0.019	−0.010	**0.761**	0.348	−0.215	**0.772**
Items: conspiracy narratives						
n01	**0.608**	−0.277		**0.775**	−0.643	
n02	**0.745**	0.083		**0.696**	−0.366	
n03	**0.902**	0.145		**0.814**	−0.397	
n04	**0.671**	−0.114		**0.740**	−0.518	
n05	0.100	**−0.848**		0.610	**−0.909**	
n06	0.045	**−0.907**		0.590	**−0.934**	
n07	−0.040	**−0.966**		0.541	**−0.942**	
n08	**0.753**	−0.107		**0.817**	−0.559	
n09	**0.643**	−0.208		**0.768**	−0.595	

Rotation method: oblimin with Kaiser normalisation. Component correlations are − 0.240 (1, 2), 0.429 (1, 3), and − 0.262 (2, 3); negative correlations are due to the negative factor loadings for the second component. Main loadings are highlighted in bold.

Furthermore, principal component analyses were conducted separately for each construct (oblimin rotation with Kaiser normalisation, extraction based on eigenvalues >1). Only one factor each emerged for religiosity as well as conspiracy mentality. The conspiracy narratives items could be divided into two factors – the first comprising six items and the second comprising three items (refer to Table 2). Cautiously interpreted, these components might be seen as representing (1) narratives focusing on the exertion of power or an assessment of the virus as being man-made vs. representing (2) a focus on disinformation or downplaying the pandemic’s danger. For the sake of brevity, we will in the remainder of this text refer to the first factor as “power” and the second factor as “disinformation.”

**Figure 1:**
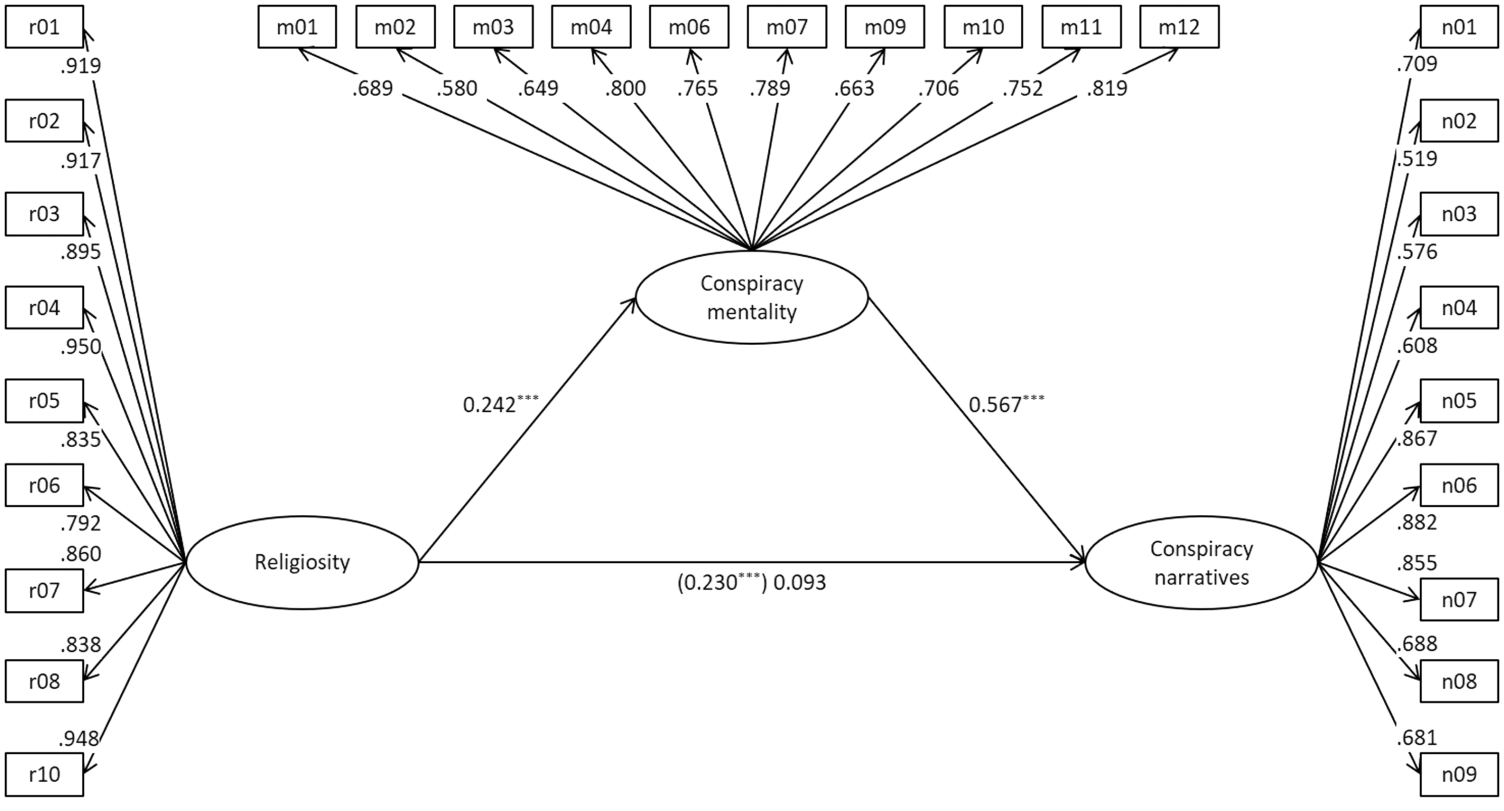
Prediction of conspiracy narratives (standardised coefficients). For all factor loadings *p* < 0.001. ***p < 0.001. Indirect effect = 0.137, *p* < 0.001.

Using Mplus 8 (Muthén and Muthén, 2017), we tested our mediation model as specified in Figure 1, i.e., with religiosity, conspiracy mentality, and conspiracy narratives as latent variables, and additionally included age, gender (0 = male, 1 = female), and education (without academic degree = 0, with academic degree = 1) as manifest control variables, employing a maximum likelihood estimator with robust standard errors (MLR). As hypothesised, conspiracy mentality mediated religiosity’s relation with conspiracy narratives as indicated by a highly significant indirect effect (here and in the remainder of the text, standardised path coefficients are reported): β = 0.137, *p* < 0.001. *RMSEA* (0.058) and *SRMR* (0.071) indicate a good model fit; *CFI* (0.876) and *TLI* (0.866) approach the respective threshold of 0.90 for an acceptable fit (Brown, 2006); χ^2^(425) = 1,292.634, *p* < 0.001; *AIC* = 42,101.12.^1^ Regarding the control variables, only the prediction of conspiracy mentality by education (β = −0.170, *p* < 0.001) reached statistical significance. The prediction of conspiracy narratives by age approached significance (β = −0.070, *p* = 0.059).

**Figure 2:**
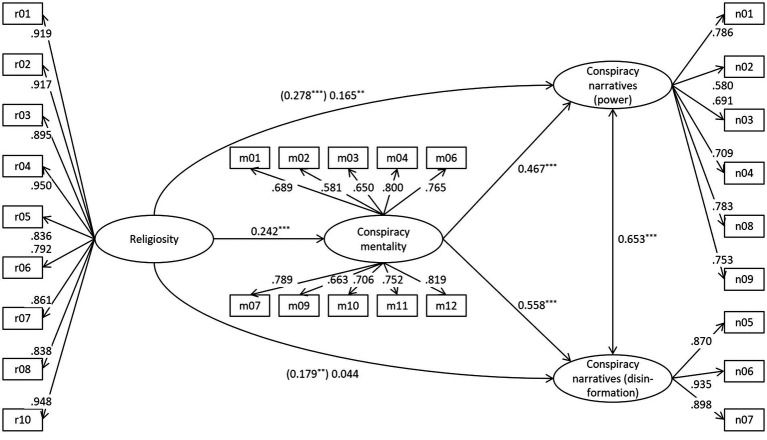
Prediction of conspiracy narratives: two factors (standardised coefficients). For all factor loadings *p* < 0.001. **p < 0.01 ***p < 0.001. Indirect effect (power) = 0.113, *p* < 0.001; indirect effect (disinformation) = 0.135, *p* < 0.001.

Additionally, we reran the analysis with the two separate factors of conspiracy narratives as criteria (Figure 2), again with the three socio-demographic variables as controls. Conspiracy mentality mediated the relation of religiosity and both facets of conspiracy narratives as indicated by highly significant (*p* < 0.001 in each case) indirect effects: β = 0.113 (power), β = 0.135 (disinformation). This solution yielded even better model fit parameters: *RMSEA* = 0.050, *SRMR* = 0.064, *CFI* = 0.910, *TLI* = 0.900, χ^2^(419) = 1,053.486, *p* < 0.001; *AIC* = 41,630.95.^2^ In addition to the statistical influence of education on conspiracy mentality (β = −0.170, *p* < 0.001), age significantly predicted the disinformation facet of conspiracy narratives (β = −0.082, *p* = 0.025).^3^

## 4. Discussion

Relationships between conspiracy mentality and support for conspiracy narratives as well as paranormal belief (including religiosity) have been reported in the past (Bruder et al., 2013). An integration as proposed in our mediation model is – to our knowledge – a novel approach.

Overall, the proposed model received strong empirical support: religiosity turned out to be a significant predictor of support for conspiracy narratives; this effect was significantly mediated *via* conspiracy mentality. In other words, the more religious people are, the more likely they are to believe in conspiracy narratives. This relation is explained by conspiracy mentality, implying that religiosity positively predicts conspiracy mentality which, in turn, positively predicts support of conspiracy narratives.

It is important to stress that the three theoretical concepts clearly emerged as three distinct empirical factors. Thus, it appears useful and justified to separate religiosity, conspiracy mentality, and support for conspiracy narratives in spite of their conceptual similarity.

Moreover, we consider it important to keep in mind that our operationalisation of religiosity focuses on the conviction of the existence of an intervening, almighty, omniscient god. Insofar, our model neglects other conceptualisations or facets of religion (see e.g., Wulff, 1997 or subsequent work by Duriez et al., 2000, 2007). Future research could address related questions, e.g., whether religiosity as defined in a broader sense is similarly related to conspiracy mentality and conspiracy narratives. However, religiosity as operationalised in this study appears to also be important for wider conceptualisations. Without the consensual conviction of god’s existence, for example, a defining element of the respective social identity would be missing. Furthermore, belief in god is central to religious practice, the profession of faith, e.g., is part and parcel of religious service.

Interestingly, out of the employed control variables only education played a significant role in the prediction of conspiracy mentality (higher education was related to less pronounced conspiracy mentality) and age significantly predicted the disinformation facet of conspiracy narratives. Thus, it seems reasonable to suppose that more and better education would be an effective antagonist of conspiracy mentality. Younger participants tend to more strongly endorse conspiracy narratives, particularly those revolving around disinformation. Further research could investigate potential reasons for this relation; growing up in different times may well mean resorting to different life experiences or strategies when confronted with conspiracies. Taking this thought of differences in habits or life experiences between age cohorts further, one might speculate that religiosity bears different meanings for people in different stages of life. Insofar, future research could turn to investigating such differences among cohorts and potential repercussions for our model.

Even though our data do not allow final conclusions concerning causality, we advocate the following line of argument. Religiosity is usually developed early in life as religious beliefs and practices are handed down from generation to generation (e.g., Dawkins, 2007; Smith, 2021). Religiosity, as operationalised in this study, necessarily implies convictions (e.g., of god’s existence) in the absence of proof or even in spite of convincing counterevidence (e.g., Law, 2010). Religiosity moreover implies a *selbstverschuldete Unmündigkeit* (self-imposed immaturity; Kant, 1999) insofar as responsibility for events and outcomes is shifted to a supernatural agent; explanations by (religious) authorities are not challenged but tend to be unquestioningly accepted; as Brookmyre (1999, p. 106) humorously puts it in one of his novels: “Heck, all that thinking just made things too damn complicated. Besides, we already know all the answers.” It appears plausible to assume that such an acquired *Unmündigkeit* can emulate and spill over to different (super-) natural agents. Therefore, religiosity may not only be a temporal but also a causal antecedent to conspiracy mentality. Following this line of thinking, religiosity implies or even fosters a tendency to accept unsubstantiated claims or explanations outside religion.

It may well be true that conspiracy narratives and religious teachings can be contradictory on particular issues. While, e.g., there are conspiracy narratives revolving around the coronavirus being deliberately brought into existence, the German Catholic, Protestant, and Orthodox churches consider it a hardship without fault (Bätzing et al., 2020). One might, therefore, ask whether the (mediated) relationship between religiosity and conspiracy narratives remains for such topics. Future research could shed light on this question. Given the evidence that contradictory conspiracy narratives are positively related (Wood et al., 2012), it seems likely, however, that even despite contradictions between religious teachings and conspiracy narratives their positive link would persist: logic does not appear to prevent such implausible psychological integration of convictions.

Furthermore, a simultaneous endorsement of both – religiosity and conspiracy narratives – is supposedly rather unproblematic as conspiracy narratives probably seldom directly clash with fundamental religious convictions. Thus, conspiracy narratives/mentality and religion may usually extend across separate domains. While religions often touch on questions like, e.g., the creation or the hereafter conspiracies typically revolve around more worldly matters like, e.g., a small elite holding extraordinarily large power.

Thus far, we have argued that religiosity may foster conspiracy mentality. Considering potential constructs mediating this relation, an expanded explanation emerges: if someone learns and applies critical thinking from early on, they should be less prone to take unsubstantiated claims – religious or conspiracist – “at face value, without really thinking about it” (Fisher, 2001, p. 14). The latter characterises unreflective, hence the opposite of critical thinking (Fisher, 2001). Therefore, early religious instruction might impede the development of critical thinking skills which might well be a causal antecedent for conspiracy mentality.

This supposition ties in with former research. On a general level, assuming a positive relation between intelligence and critical thinking skills, the finding of a negative relationship between intelligence and religious belief (Lynn et al., 2009) lends support to our argumentation. Swami et al. (2014) further back the plausibility of our claim reporting a negative relationship between belief in conspiracy theories and a rational thinking style (study 1) and experimentally demonstrating that analytic thinking reduces such a belief (studies 2–4). Similar negative relationships between “an analytic cognitive style, defined as a propensity to engage in effortful reasoning,” and “a tendency to subscribe to both religious and paranormal forms of supernatural belief” are reported by Pennycook et al. (2012, p. 343). Given these relationships, we suggest future research regarding religiosity and conspiracy narratives should include critical thinking as additional construct.

While the three theoretical constructs were very well represented in the data, at closer (i.e., separate) inspection, the support of conspiracy narratives divided into two factors. Earlier, we suggested that these factors may represent exertion of power (or viewing the virus as being man-made) vs. disinformation (or downplaying the danger). Even though the topic – COVID-19 – of the narratives was intended to be just an exemplary case of conspiracy narratives in general, it seems worthwhile to examine in future research whether these factors are stable across time and settings and if they serve different psychological functions.

Summing up, we found solid evidence for the proposed mediation model: religiosity predicts support of conspiracy narratives, and this link is mediated *via* conspiracy mentality. Therefore, we suggest the role of religiosity in the support of conspiracy narratives should not be neglected, e.g., in educational campaigns countering disinformation. It could be useful to motivate the public to dare to know (“*sapere aude,”* Kant, 1999) in general and not only in specific domains because “Those who can make you believe absurdities can make you commit atrocities” (quote attributed to Voltaire; Dawkins, 2007, p. 345). Unfortunately, this pessimistic statement appears to apply well to conspiracy narratives as those supporting these narratives tend to endorse or even perpetrate violence (Lamberty, 2020).

## Data availability statement

The raw data underlying the findings described and used to reach the conclusions of the manuscript are available via PsychArchives (https://doi.org/10.23668/psycharchives.12339).

## Ethics statement

This study was carried out in accordance with the “Berufsethische Richtlinien des Berufsverbandes Deutscher Psychologinnen und Psychologen e.V. und der Deutschen Gesellschaft für Psychologie e.V.” [professional ethics guidelines of the occupational union of German psychologists and the German society of psychology]. All subjects gave informed consent in accordance with the Declaration of Helsinki. An ethics approval was not required as per the university’s guidelines or national regulations. The research presented no discernible risk to the participants.

## Author contributions

HG wrote the original draft, conducted the analyses, and supervised the data collection. AR developed and assembled items, conducted the data collection, and reviewed and edited the manuscript. HG and AR developed the research idea, model, and hypotheses and designed the study. All authors contributed to the article and approved the submitted version.

## Funding

We acknowledge financial support by DFG within the funding programme Open Access-Publikationskosten.

## Conflict of interest

The authors declare that the research was conducted in the absence of any commercial or financial relationships that could be construed as a potential conflict of interest.

## Publisher’s note

All claims expressed in this article are solely those of the authors and do not necessarily represent those of their affiliated organizations, or those of the publisher, the editors and the reviewers. Any product that may be evaluated in this article, or claim that may be made by its manufacturer, is not guaranteed or endorsed by the publisher.

## Footnotes

^1^When predictor and mediator are switched, the model does not yield a significant indirect effect (*a × b* = 0.022; *p* = 0.157).

^2^With conspiracy mentality as predictor and religiosity as mediator, the total indirect effect for the power aspect of conspiracy narratives (*a × b* = 0.039) is significant (*p* = 0.029), while the indirect effect for the disinformation aspect (*a × b* = 0.010) is not (*p* = 0.403).

^3^In a further analysis, we entered information about religious affiliation (none = 0; Christian = 1) as an additional control variable in both models. While the indirect paths’ coefficients shrank somewhat, the mediation patterns stayed the same and remained significant. Religious affiliation was unrelated to conspiracy mentality but significantly predicted the power aspect of conspiracy narratives in the second model (β = −0.164, *p* = 0.006), indicating stronger support for these items among non-affiliated participants.
