# Supplementary material for: Conviction in the absence of proof: Conspiracy mentality mediates religiosity’s relationship with support for COVID-19 conspiracy narratives
Source: Front Psychol. 2023 Feb 16;14:1026144. doi: 10.3389/fpsyg.2023.1026144 (PMC9996749; doi:10.3389/fpsyg.2023.1026144)
Supplement: Supplementary file 1 [file Table_1.pdf]

Supplementary material (Grabow & Rock, 2023)

Items

	German	English
Religiosity		
r01	Gott existiert.	God exists.
r02	Gott ist allmächtig.	God is almighty.
r03	Gott ist allwissend.	God is omniscient.
r04	Ich habe Vertrauen in Gottes Entscheidungen.	I have confidence in god's decisions.
r05	Gott lenkt die Geschehnisse auf der Welt.	God directs events in the world.
r06	Einen Zufall gibt es nicht – Gott lenkt.	There is no such thing as chance – god directs.
r07	Mein Glaube an Gott reicht mir als Beweis seiner Existenz.	My belief in god is enough for me as proof of his existence.
r08	Es gibt (göttliche) Wunder.	There are (divine) miracles.
r09[a]	Es gibt ein Leben nach dem Tod.	There is life after death.
r10	Ich glaube an Gott.	I believe in god.
Conspiracy mentality[b]		
m01	Es geschehen sehr viele wichtige Dinge in der Welt, über die die Öffentlichkeit nie informiert wird.	There are very many important things happening in the world that the public is never informed about.
m02	Die da oben machen ja eh was sie wollen.	They do what they want up there anyway.
m03	Ein paar mächtige Personengruppen bestimmen über das Schicksal von Millionen von Menschen.	A few powerful groups of people determine the fate of millions of people.
m04	Es gibt geheime Organisationen, die großen Einfluss auf politische Entscheidungen haben.	There are secret organizations that have great influence on political decisions.
m05[c]	Die verschiedenen in den Medien zirkulierenden Verschwörungstheorien halte ich für ausgemachten Blödsinn.	I consider the various conspiracy theories circulating in the media to be utter nonsense.
m06	Politiker und andere Führungspersönlichkeiten sind nur Marionetten der dahinterstehenden Mächte.	Politicians and other leaders are only puppets of the powers behind them.
m07	Die meisten Menschen erkennen nicht, in welchem Ausmaß unser Leben durch Verschwörungen bestimmt wird, die im Geheimen ausgeheckt werden.	Most people do not realize the extent to which our lives are determined by conspiracies hatched in secret.
m08[c]	Es gibt keinen vernünftigen Grund, Regierungen, Geheimdiensten oder Medien zu misstrauen.	There is no reasonable reason to distrust governments, intelligence agencies, or the media.

m09	Die internationalen Geheimdienste mischen viel mehr in alltäglichen Dingen mit, als man denkt.	The international intelligence agencies meddle in everyday matters much more than one might think.
m10	Geheime Organisationen können Leute psychisch so manipulieren, dass diese nicht wissen, dass ihr Leben von außen bestimmt wird.	Secret organizations can psychologically manipulate people so that they do not know that their lives are being determined from the outside.
m11	Es gibt bestimmte politische Zirkel, die geheime Pläne verfolgen und sehr viel Einfluss haben.	There are certain political circles that pursue secret plans and have a great deal of influence.
m12	Die meisten Menschen machen sich keine Vorstellung davon, wie sehr unser Leben bestimmt wird von im Geheimen geschmiedeten Plänen.	Most people have no idea how much our lives are determined by plans forged in secret.
Covid narratives		
n01	Bill Gates hat das Corona-Virus entwickelt, um mit einer Impfkampagne den Menschen zu schaden.	Bill Gates developed the Corona virus to harm people with a vaccination campaign.
n02	Das Corona-Virus ist eine Biowaffe der Asiaten.	The corona virus is a bioweapon of the Asians.
n03	Das Corona-Virus ist eine Biowaffe der US-Amerikaner.	The Corona virus is a US bioweapon.
n04	Das Corona-Virus existiert nicht, es ist eine Erfindung der Regierung, um unsere Grundrechte einzuschränken.	The Corona virus does not exist, it is a government invention to restrict our fundamental rights.
n05	Das Corona-Virus existiert schon lange; es ist ungefährlicher als die saisonale Grippe. Die Maßnahmen der Regierungen sind Panikmache, um unsere Grundrechte einzuschränken.	The Corona virus has been around for a long time; it is less dangerous than seasonal flu. Governments' actions are scare tactics to restrict our fundamental rights.
n06[d]	Das Corona-Virus wird absichtlich als gefährlich dargestellt, um die Öffentlichkeit in die Irre zu führen.	The Corona virus is deliberately presented as dangerous in order to mislead the public.
n07[d]	Experten führen uns absichtlich zu ihrem eigenen Vorteil in die Irre, obwohl das Virus nicht schlimmer als eine Grippe ist.	Experts deliberately mislead us for their own benefit, even though the virus is no worse than flu.
n08[d]	Corona wurde absichtlich in die Welt gebracht, um die Bevölkerung zu reduzieren.	Corona was deliberately brought into the world to reduce the population.
n09[d]	Dunkle Mächte wollen das Virus benutzen, um die Welt zu beherrschen.	Dark forces want to use the virus to dominate the world.

Notes.

[a] Item r09 was dropped from the scale.

[b] Conspiracy mentality scale (Imhoff & Bruder, 2014).

[c] Reverse coded. The items m05 and m08 were dropped from the conspiracy mentality scale as their inclusion would have decreased Cronbach's α by .012.

[d] Taken from Imhoff and Lamberty (2020).
